# Leucine zipper downregulated in cancer 1 may serve as a favorable prognostic biomarker by influencing proliferation, colony formation, cell cycle, apoptosis, and migration ability in hepatocellular carcinoma

**DOI:** 10.3389/fgene.2022.900951

**Published:** 2022-07-25

**Authors:** Huaping Chen, Siyuan Chen, Chen Chen, Aifeng Li, Zhixiao Wei

**Affiliations:** ^1^ Department of Clinical Laboratory, First Affiliated Hospital of Guangxi Medical University, Nanning, GX, China; ^2^ Department of Nuclear Medicine, First Affiliated Hospital of Guangxi Medical University, Nanning, GX, China

**Keywords:** biomarker, prognosis, hepatocellular carcinoma, tumor suppressor gene, LODC1

## Abstract

**Aims**: Leucine zipper downregulated in cancer 1 (LDOC1) inhibits tumor growth in several cancers. However, the expression and function of LDOC1 in hepatocellular carcinoma (HCC) remain unknown. In this study, we aimed to investigate how LDOC1 influenced tumor progression and the biological functions of HCC.

**Methods**: The transcription levels of LDOC1 were determined using the GEPIA and UALCAN online databases and a real-time polymerase chain reaction. Western blot and immunohistochemistry were used to validate the protein levels of LDOC1. The online Kaplan-Meier Plotter was applied for survival analysis. Then lentivirus transfection was used to construct LDOC1 exogenous overexpression cell lines. Proliferation, clone formation, cell cycle, apoptosis, and migration assays were performed with the LDOC1-upregulated Huh7 and Hep3B cell lines. The phosphorylated and total levels of AKT and mTOR were determined using a Western blot to explore the potential molecular mechanism of LDOC1.

**Results**: In the GEPIA and UALCAN analyses, LDOC1 was lowly expressed in tumors, had high expression in normal tissue samples (*p* < 0.05), and negatively correlated with tumor grade progression. The down-regulation of LDOC1 in HCC was validated with real-time polymerase chain reaction, Western blot, and immunohistochemistry (all *p* < 0.05). LDOC1 transcription levels were negatively associated with overall, progression-free, recurrence-free, and disease-specific survival (all *p* < 0.05). The functional experiments suggested that the overexpression of LDOC1 contributed to increased G1 and G2 stages in Huh7, while increased G2 stage in Hep3B, and decreased cell proliferation, clone formation, and migration, as well as increased the apoptosis rate compared with the control group (all *p* < 0.05). Furthermore, LDOC1 up-regulation reduced the p-AKT/AKT and p-mTOR/mTOR, which indicates an inactivation of the AKT/mTOR pathway.

**Conclusion**: The tumor-suppressor LDOC1 varied in HCC and non-HCC tissues, which can serve as a candidate prognostic biomarker. LDOC1 influenced survival by affecting proliferation, colony formation, cell cycle, apoptosis, and migration ability, which might be attributed to the AKT/mTOR inhibition in HCC.

## 1 Introduction

Liver cancer is one of the leading causes of cancer death and one of the highest disease burdens in many countries (Siegel et al., 2021). The most common primary liver cancer is hepatocellular carcinoma (HCC), accounting for about 90% of cases, followed by cholangiocarcinoma (Siegel et al., 2021). Hepatocellular carcinoma mainly occurs in patients with underlying chronic hepatitis B virus (HBV) or hepatitis C virus (HCV) infection, excessive alcohol drinking, food contamination *via* Aspergillus spp., and aflatoxin B_1_, or non-alcoholic fatty liver disease. In Asia, HBV is the leading risk factor for HCC, whereas HCV and alcoholic and non-alcoholic liver disease are the major causes of HCC in Europe and North America. Thus, many causes involving genetic and environmental factors could affect the development of HCC. The overall outcomes of HCC patients usually depend on neoplasm staging and the underlying liver function. Although the overall survival rate of HCC patients has increased in the past decade, the overall prognosis remains poor. Therefore, it is very important to understand the complex molecular mechanisms of HCC pathogenesis which may lead to future targeted therapies.

In 1999, Nagasaki et al. firstly found that the leucine zipper downregulated in cancer-1 (LDOC1) as a low-expressed gene was identified in several tumor cells (Nagasaki et al., 1999). LDOC1 encodes a protein with a leucine zipper-like motif and an SH3-binding domain, which may regulate intracellular signal transduction and gene transcription. Downregulation of LDOC1 was discovered in various tumor tissues, such as the lung (Lee et al., 2015; Lee et al., 2019), colorectum (Jiang et al., 2019), cervix (Buchholtz et al., 2013; Chen and Wang, 2019), vulva (Wanka et al., 2020), pancreas (Nagasaki et al., 2003), and papillary thyroid (Zhao et al., 2015). These studies have demonstrated that a higher level of LDOC1 decreases the proliferation of cancer cells or is associated with a favorable prognosis (Nagasaki et al., 2003; Zhao et al., 2015; Jiang et al., 2019; Wanka et al., 2020). Numerous studies demonstrated that the LDOC1 gene inhibits ligand-induced NF-κB activity in oral squamous cell carcinoma (Lee et al., 2013), papillary thyroid carcinoma (Zhao et al., 2015), and pancreatic cancer cells (Nagasaki et al., 2003). In addition, AKT/mTOR signaling was also an essential pathway in regulating cell proliferation and migration (Shao et al., 2022). However, to date, no information about the expression and function of LDOC1 in liver cancer exists. Therefore, we analyzed the expression of LDOC1 in databases and tissue samples, as well as the role of LDOC1 in the HCC prognosis, including overall survival (OS), progression-free survival (PFS), recurrence-free survival (RFS), and disease-specific survival (DSS). Leucine zipper downregulated in cancer-1 was exogenously overexpressed to explore its potential functions, including cell proliferation, colony formation, cell cycle, apoptosis, and migration in HCC cell lines. Furthermore, the phosphorylated levels of AKT and mTOR and their total levels were determined with a western blot to explore the potential molecular mechanism of LDOC1.

## 2 Materials and methods

### 2.1 Gene expression and network construction

The GEPIA (Li et al., 2021) and UALCAN (Chandrashekar et al., 2017) databases were used to analyze the gene expression, transcript level, and tumor grades. Gene co-expression and protein-protein interaction networks were established using the online GeneMANIA website (Franz et al., 2018) and the Search Tool for the Retrieval of Interacting Genes database (STRING) website (Szklarczyk et al., 2019), respectively.

### 2.2 Tissues samples and expression validation

The Ethics Committee of the First Affiliated Hospital of Guangxi Medical University approved this research and written informed consent was obtained from all patients. We collected 54 pairs of HCC tissues and adjacent normal tissues from the First Affiliated Hospital of Guangxi Medical University (January 2019–January 2020). The included patients with HCC were determined by postoperative pathology. Patients will be excluded if they fulfill the following criteria. (1) Patients with a complicated or previous history of other malignant tumors or liver metastasis of other tumors, (2) preoperative particular antitumor therapy, including radiofrequency ablation, intervention, etc., (3) patients who died during the perioperative period, (4) clinical information deficiency, and (5) patients with distant metastasis. Fresh tissues were stored with RNA-EZ Reagents D RNA-Be-Locker A (Sangon, cat. no. B644171) at −80°C. Then the RNA was extracted, which was amplified with reverse transcription-polymerase chain reaction (RT-PCR), as per the prescribed protocols. The RT-PCR primers were designed and synthesized by Sangon Biotech (Shanghai) Co., Ltd., and the sequences were: LDOC1, forward, 5'-ATG​AAG​GTG​GCA​TTC​CTA​ATC​A-3', and reverse, 5'-AAT​CAT​CCT​CCT​CTT​CTT​CGT​C-3'; and GAPDH, forward, 5'-TGA​CTT​CAA​CAG​CGA​CAC​CCA-3' and reverse, 5'-CAC​CCT​GTT​GCT​GTA​GCC​AAA-3'. Another tissue chip, including 31 pairs of HCC and normal tissue purchased from Nanning Gold Tech Biotechnology Co., Ltd., was used to detect the levels of proteins by the subsequent immunohistochemistry (IHC) protocols. The LDOC1 antibody (cat. no. PA5-50438) for IHC was obtained from Thermo Fisher Scientific. Three professional pathologists independently determined the positive or negative results of IHC. The histochemistry score (H-score) was used to quantify the overall positive intensity of IHC. Besides, another four pairs of fresh tissues were collected from the First Affiliated Hospital of Guangxi Medical University (June 2021) for protein extraction, and the protein was immediately extracted for subsequent Western blot as described (Qin et al., 2013). The LDOC1 antibody (cat. no. 10113-2-AP) for the Western blot was purchased from Proteintech. The antibodies of mTOR (#2983), p-mTOR (#5536), AKT (#4691), and p-AKT (#4060) were obtained from Cell Signaling TECHNOLOGY. The integrated density results of Western blot were quantified by Image J software.

### 2.3 Survival analysis

Survival analysis as OS, PFS, RFS, and DSS with LDOC1 was conducted using the online Kaplan-Meier Plotter (Menyhart et al., 2018). The LDOC1-23641, as the unique RNA-Seq ID, was used for further analysis. Stratified analysis of OS was conducted based on several prognostic factors, such as tumor stages, AJCC_T, alcohol consumption, and hepatitis virus infection.

### 2.4 Cell culture

Huh7 and Hep3B cell lines, purchased from the Cell Bank of the Chinese Academy of Sciences, were applied for the functional experiments. The cell lines were cultured in Dulbecco’s Modified Eagle’s Medium (Gibco, cat. no. C11995500BT) with 10% fetal bovine serum (Gibco, cat. no. 10099141C), with 5% CO_2_ in a 37°C incubator. Mycoplasma contamination was detected monthly.

### 2.5 Cell transfection and puromycin screening

The recombinant lentiviral vector (Lv-LDOC1) and the empty vector (Lv-NC) were design and synthesize from Guangzhou iGene Biotechnology Co., Ltd. With the assistance of 5 μg/ml polybrene (Beyotime Biotechnology, cat. no. C0351), the Huh7 and Hep3B cells were transfected with Lv-NC or Lv-LDOC1 vectors, as previously (Jiang et al., 2019). Puromycin resistance is marked into the plasmid vector, then, we selected stable cell lines by adding 5 μg/ml of puromycin (Solarbio, cat. no. P8230) to the complete medium at 37°C incubator for 3–5 weeks after 48 h of intervention. The LDOC1 expression levels were then examined by RT-PCR and Western blot in the Lv-NC and Lv-LDOC1groups.

### 2.6 Cell proliferation and colony formation

Cell Counting Kit-8 (MedChemExpress, cat. no. HY-K0301) was used to detect cell proliferation. The stably transfected Huh7 and Hep3B cell lines were seeded in 96-well plates at a 3,500 cells/well density. CCK8 reagent was added to each well, and cell proliferation was assessed at 0, 24, 48, and 72 h by detecting the 450 nm absorbance with a microplate reader (Thermo Fisher Scientific Co., Ltd.).

Cells were seeded to 6-well plates and cultured 10–14 days. Next, the cells were fixed with 4% paraformaldehyde (Solarbio, cat. no. P1110) and stained with 0.5% crystal violet (Solarbio, cat. no. G1065). We took pictures of the wells and three different people counted the number of colonies.

### 2.7 Cell cycle and apoptosis assays

Huh7 and Hep3B cell lines were interfered for 48 h and subsequently digested with 0.25% EDTA-free trypsin for the cell cycle assay. The cells were, respectively, washed with pre-chilled PBS twice and then fixed in 75% pre-chilled ethanol for 24 h at 20°C. Next, these cells were stained with propidium iodide (PI, BD Biosciences, cat. no. 550825).

Transfected Huh7 and Hep3B cells were trypsinized with 0.25% EDTA-free and suspended in PBS buffer for cell apoptosis. An Annexin V-Allophycocyanin (APC)/7-Amino-Actinomycin D (7-AAD) kit (MultiSciences, cat. no. 70-AT105-30) stained the cells in the dark.

Flow cytometry (BD Biosciences) determined cell cycle distribution and apoptosis and analyzed using FlowJo Version 10 software.

### 2.8 Cell migration assays

Cell migration was determined using wound-healing assays. After 48 h of transfection, cells were resuspended and placed in six-well plates. We measured the migration distances of cells at 0 and 48 h.

### 2.9 Statistical analysis

Statistical analyses were performed with SPSS24.0 (SPSS Inc., Chicago, Illinois, United States). GraphPad Prism software version 8.0 (GraphPad Software, Inc., La Jolla, California, United States) was used for the line plots, histograms, and scatter plots. Data are presented as mean ± standard deviation. Differences between the two groups were analyzed using Student’s t-test. *p* < 0.05 was considered a statistically significant difference.

## 3 Results

### 3.1 Gene expression and interaction network construction

**FIGURE 1:**
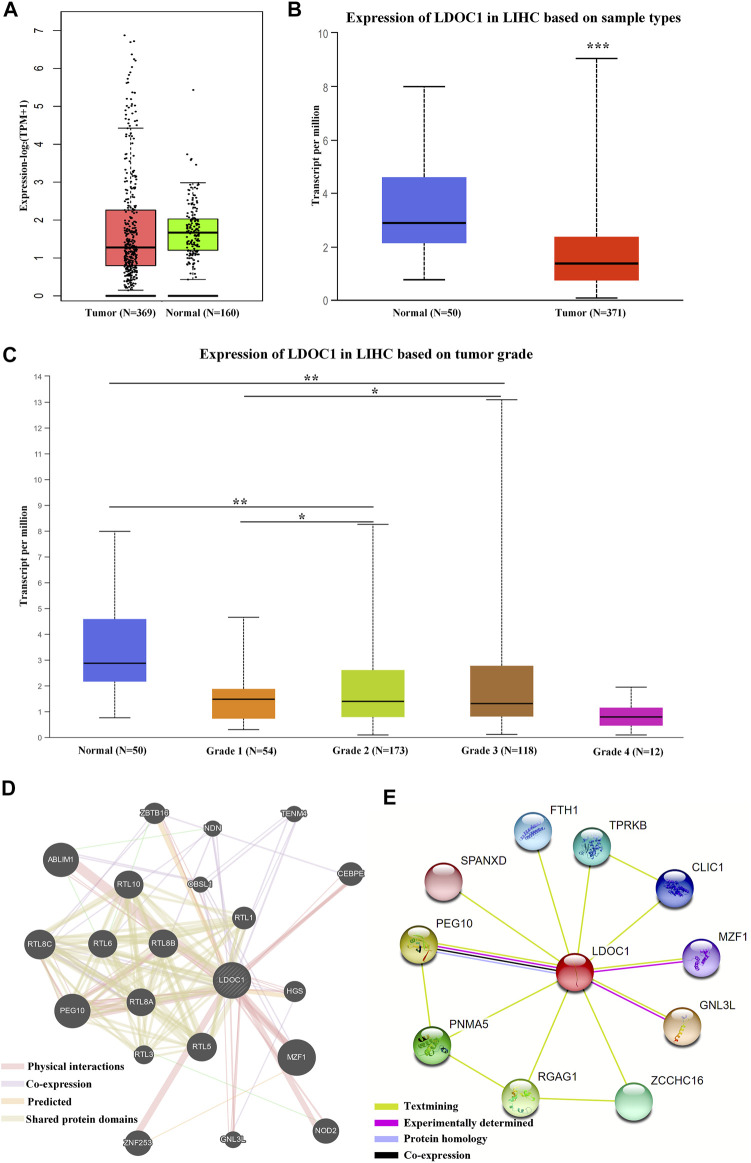
Expression levels and interaction networks of LDOC1. **(A)** Expression levels of LDOC1 in tumor and normal tissues. **(B)** Transcript levels of LDOC1 in tumor and normal tissues. **(C)** Expression levels of LDOC1 in different grades. **(D)** interaction network for LDOC1 and other genes. **(E)** Interaction network for LDOC1 and other proteins. *p < 0.05, **p < 0.01.

LDOC1 showed low expression in HCC tumor tissues (Figures 1A,B). The expression levels of LDOC1 were lower with tumor grade progression (Figure 1C). Gene-genes interaction analysis showed that LDOC1 was co-expressed with PEG10, RTL8C, RTL5, TENM4, NDN, and OBSL1; shared protein domains with PEG10, RTL8C, RTL5, RTL8B, RTL10, RTL6, RTL8A, RTL1, and RTL3; physically interacted with MZF1, PEG10, ABLIM1, ZNF253, NOD2, CEBPE, ZBTB16, HGS, and GNL3L (Figure 1D). Besides, protein-protein interaction analysis suggested that the LDOC1 protein interacted with PEG10, GNL3L, MZF1, PNMA5, RGAG1, ZCCHC16, TPRKB, CLIC1, SPANXD, and FTH1 (Figure 1E).

### 3.2 Leucine zipper downregulated in cancer 1 expression validation

**FIGURE 2:**
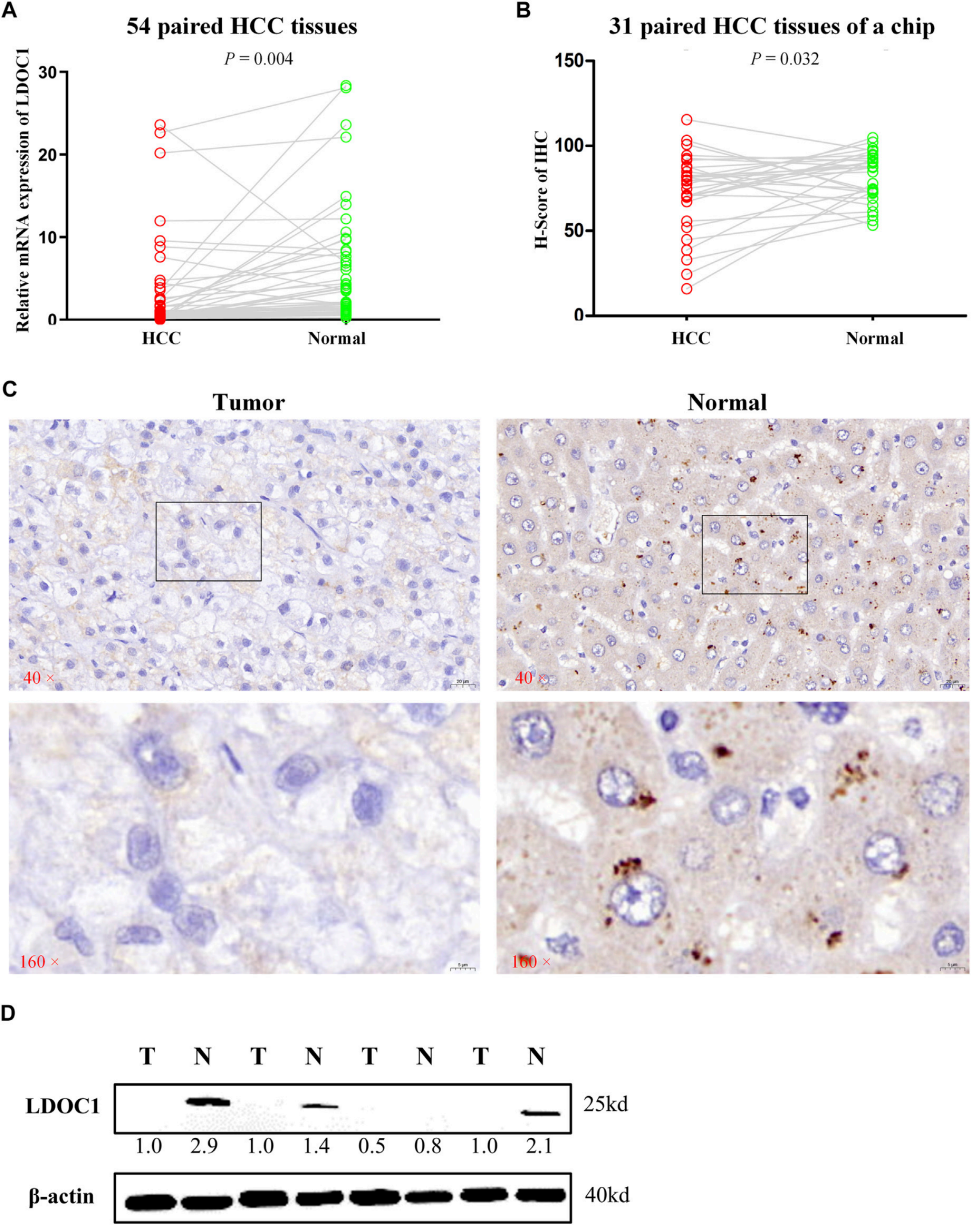
Validation experiments of LDOC1 expression. **(A)** LDOC1 mRNA levels in 54 paired HCC tissues. **(B,C)** LDOC1 protein levels in 31 paired HCC tissues of a chip *via* immunohistochemical. **(D)** LDOC1 protein levels in four paired HCC tissues *via* Western blot.

LDOC1 transcript levels were significantly downregulated in 54 HCC tissues compared to normal liver tissues (*p* = 0.004, Figure 2A). For the IHC results, only nine of 31 HCC tumor tissue showed medium and high expression of LDOC1, whereas 24 of 31 paracancerous tissues showed significant positive LDOC1. The results of the H-score analysis also demonstrated that compared with paired normal tissues, the protein expression levels of LDOC1 in HCC tissues were significantly decreased (*n* = 31, *p* = 0.032, Figures 2B,C). Furthermore, the results of a Western blot for four pairs of fresh HCC tissues also showed that the LDOC1 protein level was downregulated in HCC tissues (Figure 2D).

### 3.3 Survival analysis of leucine zipper downregulated in cancer 1

**FIGURE 3:**
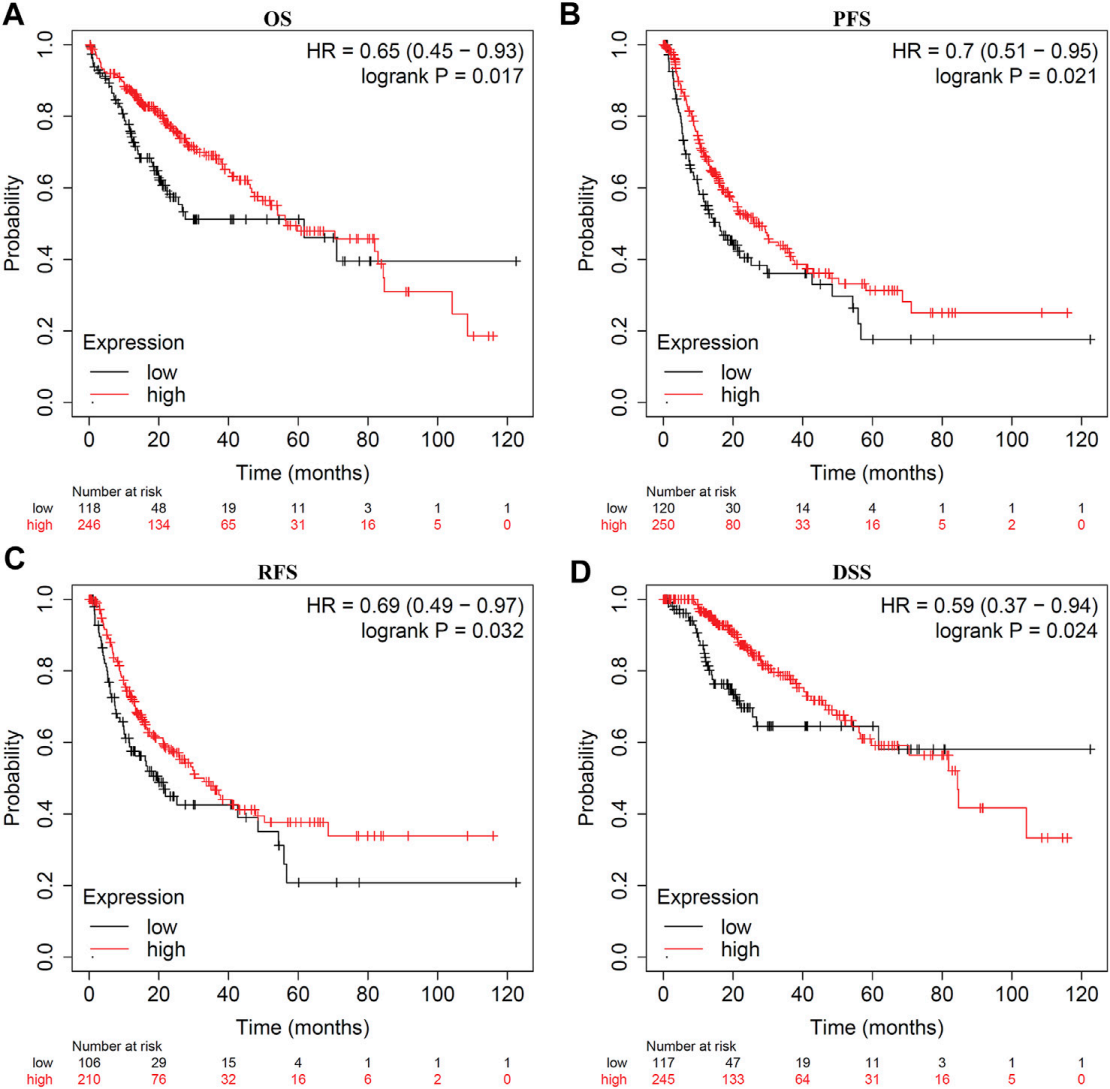
Kaplan-Meier plots for LDOC1 and overall survival (OS), progression-free survival (PFS), recurrence-free survival (RFS), and disease-specific survival (DSS). **(A)** Kaplan-Meier plot of LDOC1 and overall survival. **(B)** Kaplan-Meier plot of LDOC1 and progression-free survival. **(C)** Kaplan-Meier plot of LDOC1 and recurrence-free survival. **(D)** Kaplan-Meier plot of LDOC1 and disease-specific survival.

**FIGURE 4:**
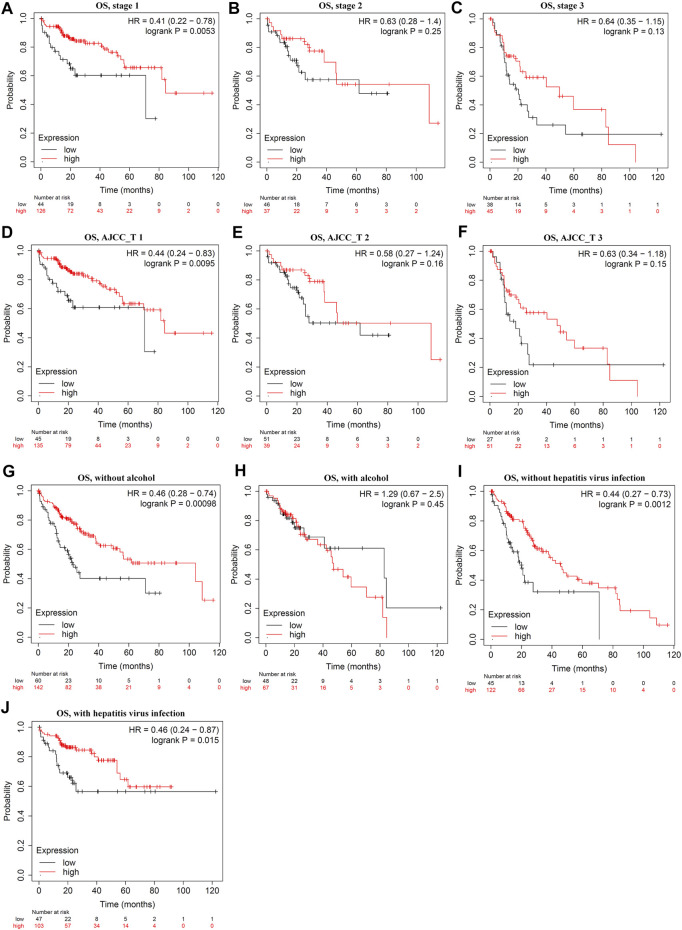
Stratified analysis of overall survival (OS) by tumor stages, ATCC stages, alcohol intake, and hepatitis infection status. **(A–C)** Stratified analysis of OS by tumor stages. **(D–F)** Stratified analysis of OS by ATCC_T. **(G,H)**, Stratified analysis of OS by alcohol intake. **(I,J)** Stratified analysis of OS by hepatitis infection.

Survival analysis indicated that LDOC1 transcriptional level was markedly associated with OS (*p* = 0.017, Figure 3A), PFS (*p* = 0.021, Figure 3B), RFS (*p* = 0.032, Figure 3C), and DSS (*p* = 0.024, Figure 3D). Stratified analysis by stages, AJCC_T, alcohol, and hepatitis virus infection suggested that OS was associated with LDOC1 in patients with Stage 1 (logrank *p* = 0.0053, Figure 4A), AJCC_T 1 (logrank *p* = 0.0095, Figure 4D), and not having alcohol intake (logrank *p* = 0.00098, Figure 4G), with or without hepatitis infection (both logrank *p* < 0.05, Figure 4I, J). However, there was no significate relationship between LDOC1 and OS in the patients with Stages 2 and 3, AJCC_T 2 and 3, and alcohol intake (all *p* > 0.05, Figures 4B,C,E,F,H).

### 3.4 Verification of leucine zipper downregulated in cancer 1 overexpression following transfection

**FIGURE 5:**
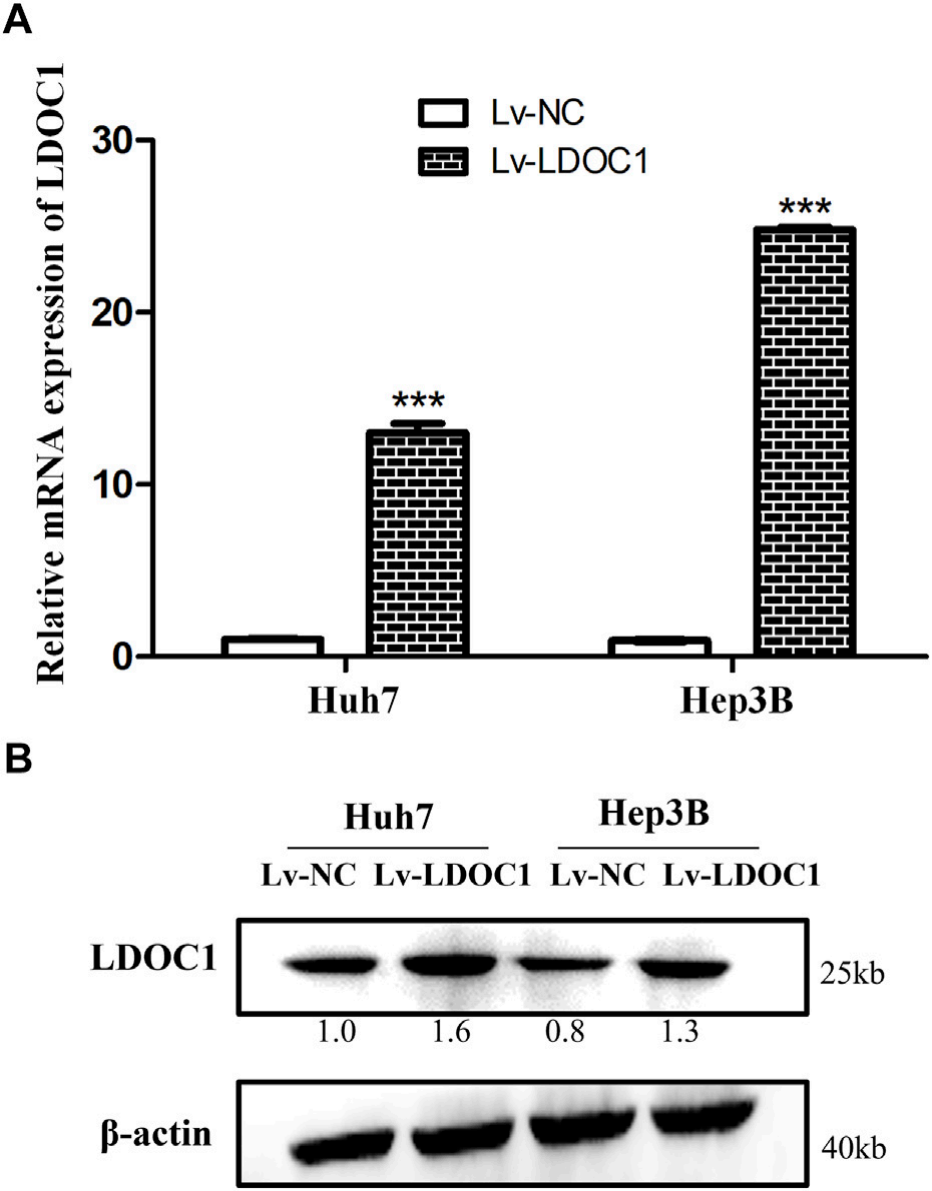
Verification of the LDCO1 overexpression after transfection. **(A)** Real-time polymerase chain reaction (RT-PCR) results. **(B)** Western blot results. ***, *p* < 0.0001.

The Huh7 and Hep3B cells were interfered with Lv-NC or Lv-LDOC1. The mRNA and protein levels of LDOC1 were detected by qRT-PCR and Western blot analysis and our results validated that the mRNA and protein expression levels of LDOC1 were significantly upregulated in the Huh7 (Lv-LDOC1) and Hep3B (Lv-LDOC1) cell lines, suggesting that we successfully constructed the exogenous LDOC1 overexpressing cell lines (all *p* < 0.05, Figures 5A,B).

### 3.5 Leucine zipper downregulated in cancer 1 inhibits hepatocellular carcinoma cell proliferation and colony formation

**FIGURE 6:**
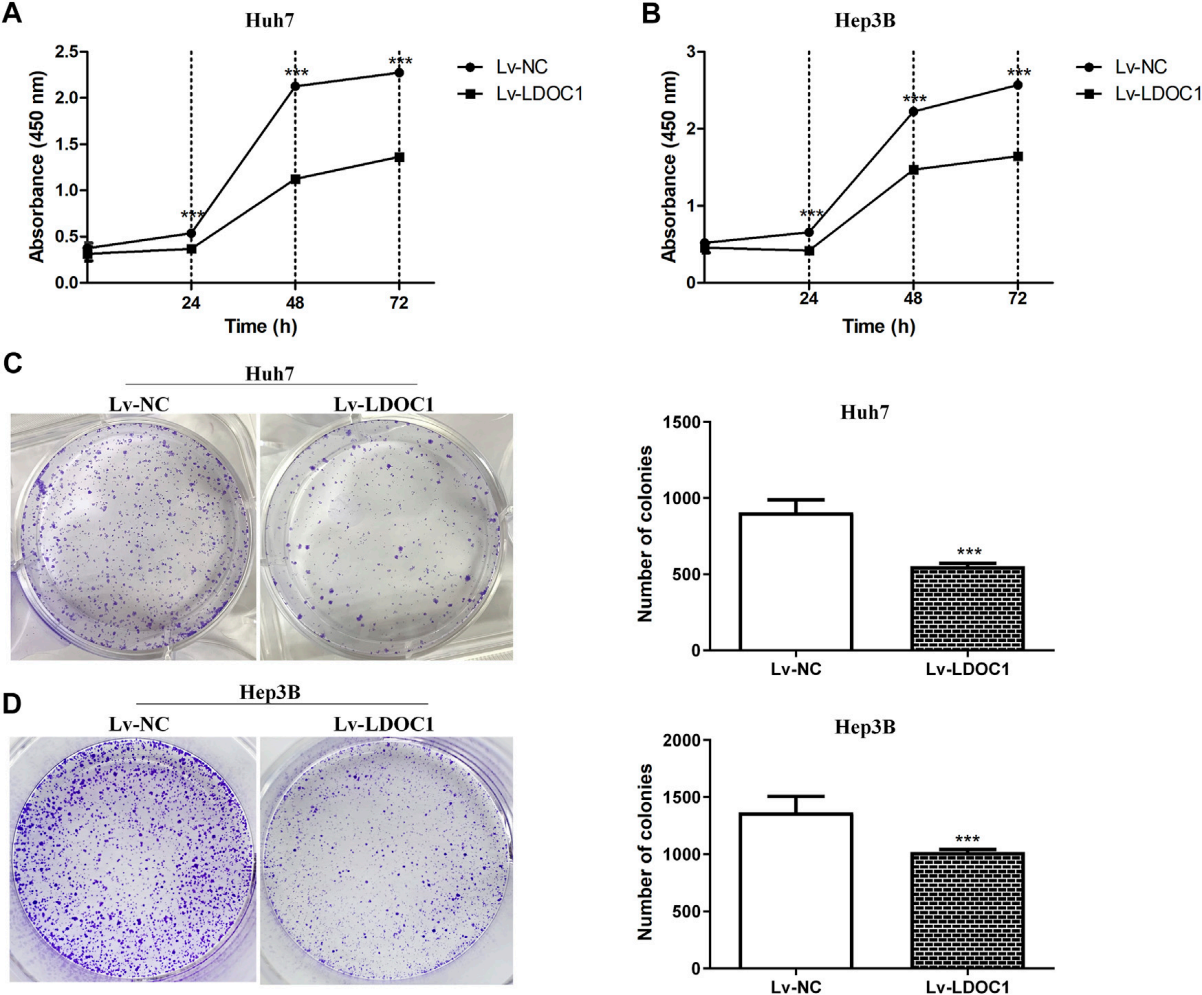
Proliferation and colony formation assays for Huh7 and Hep3B cell lines after LDOC1 overexpressing. Graphs of **(A)** Huh7 and **(B)** Hep3B cell lines for proliferation assays with Lv-NC and Lv-LDOC1 at 0, 24, 48, and 72 h. Graphs of **(C)** Huh7 and **(D)** Hep3B cell lines for colony formation assays with Lv-NC and Lv-LDOC1 at 10 days ***, *p* < 0.0001.

We determined cell proliferation using CCK8 assays and found that both Huh7 and Hep3B cell lines in the Lv-LDOC1 group showed decreased proliferation compared with the Lv-NC group (*p* < 0.01, Figures 6A,B) In addition, the colony formation assay indicated that the number of colonies formed in the Lv-LDOC1 group was significantly reduced compared to the Lv-NC group. (*p* < 0.01, Figures 6C,D).

### 3.6 Leucine zipper downregulated in cancer 1 induces cell cycle arrest and apoptosis in hepatocellular carcinoma cells

**FIGURE 7:**
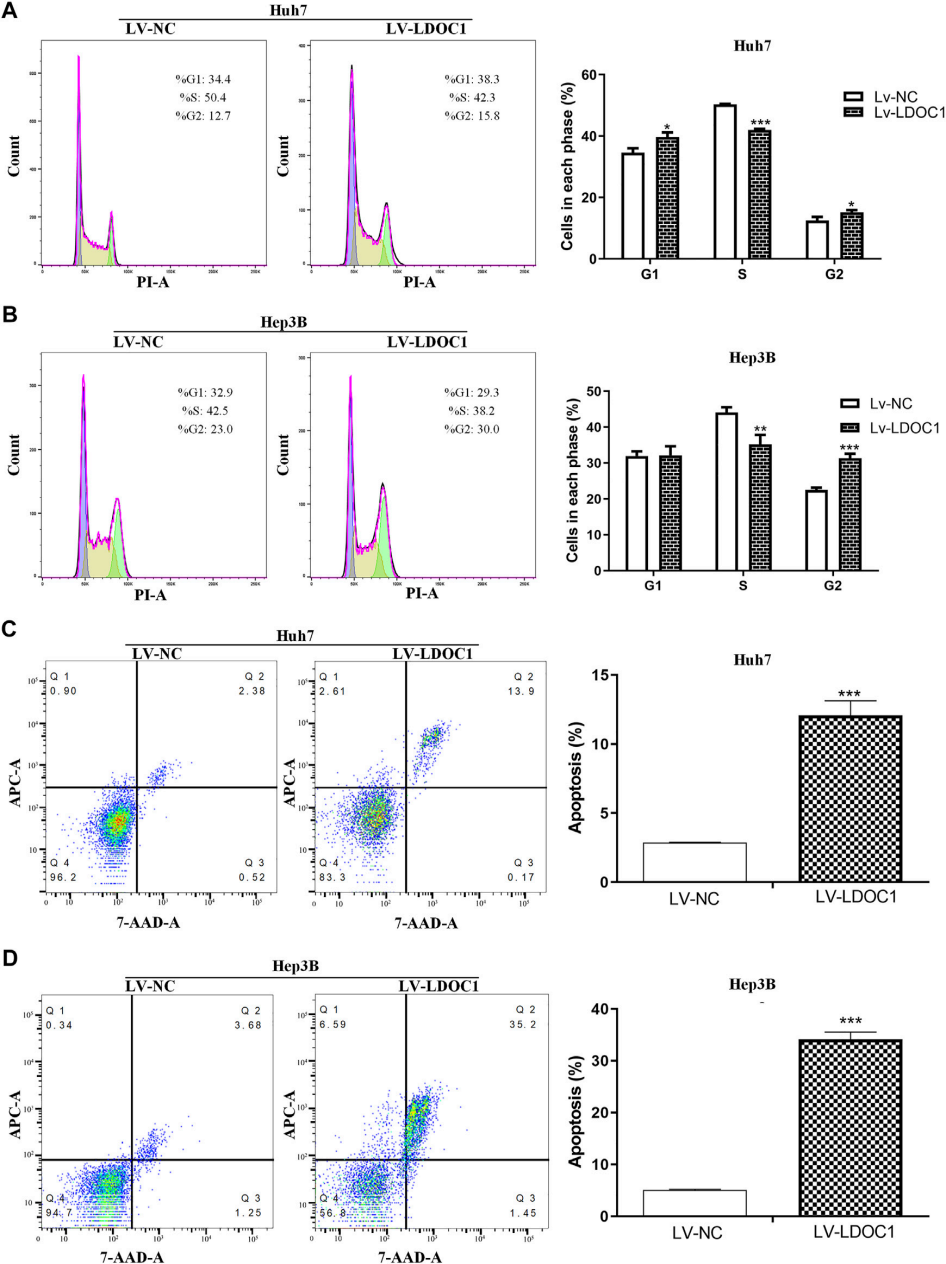
Cell cycle and apoptosis assays for Huh7 and Hep3B cell lines after LDOC1 over-expressing. From the cell-cycle distributions, **(A)** Huh7 (Lv-LDOC1) cells were significantly arrested in the G1 and G2 phases, while **(B)** Hep3B (Lv-LDOC1) cells were significantly arrested in the G2 phase. The apoptosis rates for **(C)** Huh7 and **(D)** Hep3B cell lines in the Lv-LDOC1 and Lv-NC groups. *, *p* < 0.05; **, *p* < 0.01; ***, *p* < 0.0001.

Flow cytometry was used to measure the effects of LDOC1 on the cell cycle and apoptosis of HCC cells. The results indicated that the Huh7 (Lv-LDOC1) cells were notably arrested in the G1 and G2 phases (both *p* < 0.05, Figure 7A), and Hep3B (Lv-LDCO1) cells were arrested considerably in the G2 phase (*p* < 0.001, Figure 7B). Annexin V-APC/7-AAD staining showed that the apoptosis rate of the Huh7 (Lv-LDCO1, *p* < 0.0001) and Hep3B (Lv-LDCO1, *p* < 0.0001) cells were significantly elevated compared to the Lv-NC cells (Figures 7C,D).

### 3.7 Leucine zipper downregulated in cancer 1 inhibits hepatocellular carcinoma cell migration

**FIGURE 8:**
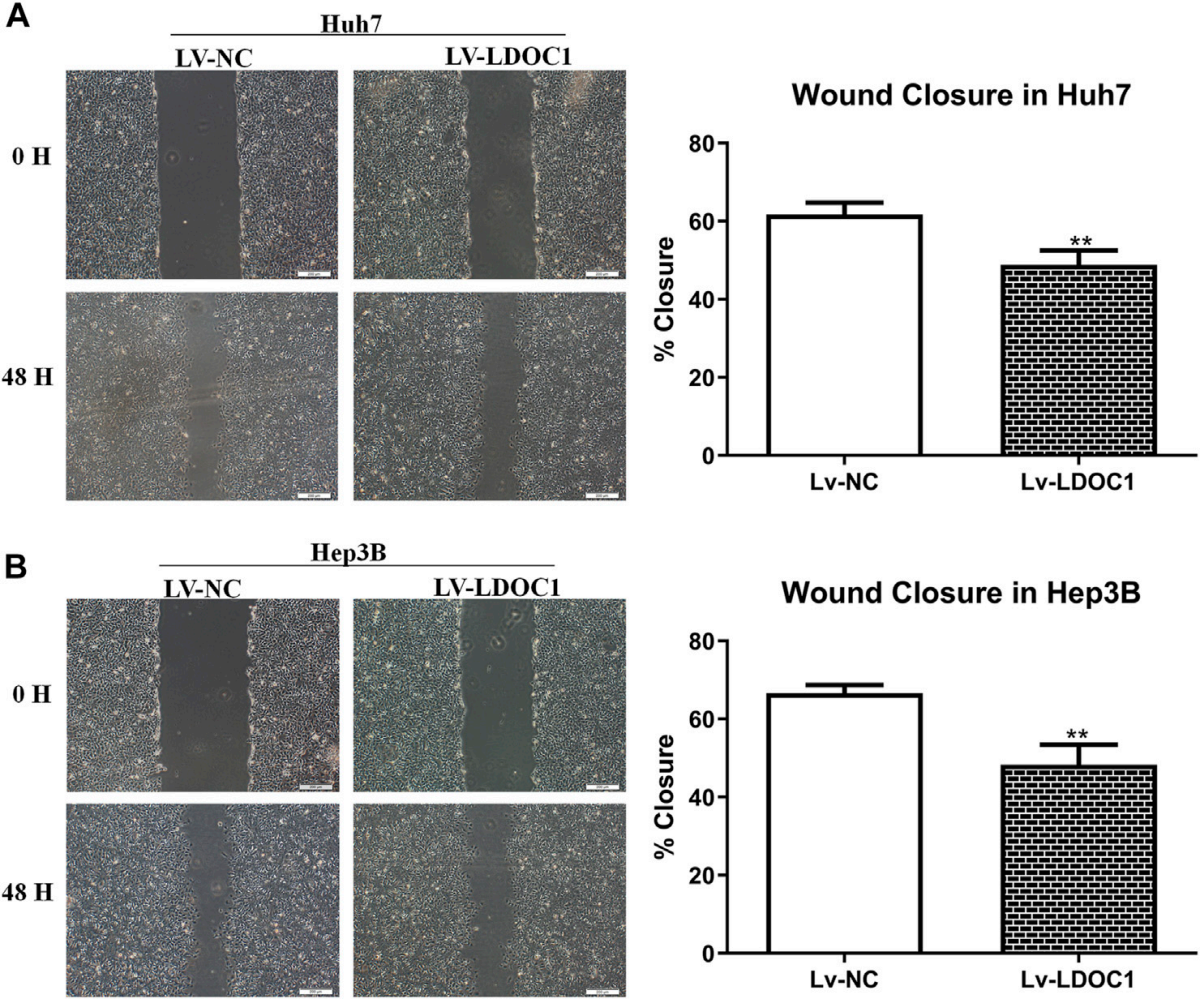
Migration ability assays for Huh7 **(A)** and Hep3B **(B)** cell lines after LDOC1 overexpressing. **, *p* < 0.01.

Wound-healing assay was used to detect cell migration ability in the Lv-NC and Lv-LDOC1 groups. Wound closure in 48 h showed significant differences compared with 0 h for both the Huh7 and Hep3B cell lines (all *p* < 0.001). Cells in the Lv-LDOC1 group decreased migration distance compared to the Lv-NC group (both *p* < 0.01; Figures 8A,B).

### 3.8 Leucine zipper downregulated in cancer 1 inhibits hepatocellular carcinoma growth by downregulating the activity of the ATK/mTOR pathway in hepatocellular carcinoma cells

**FIGURE 9:**
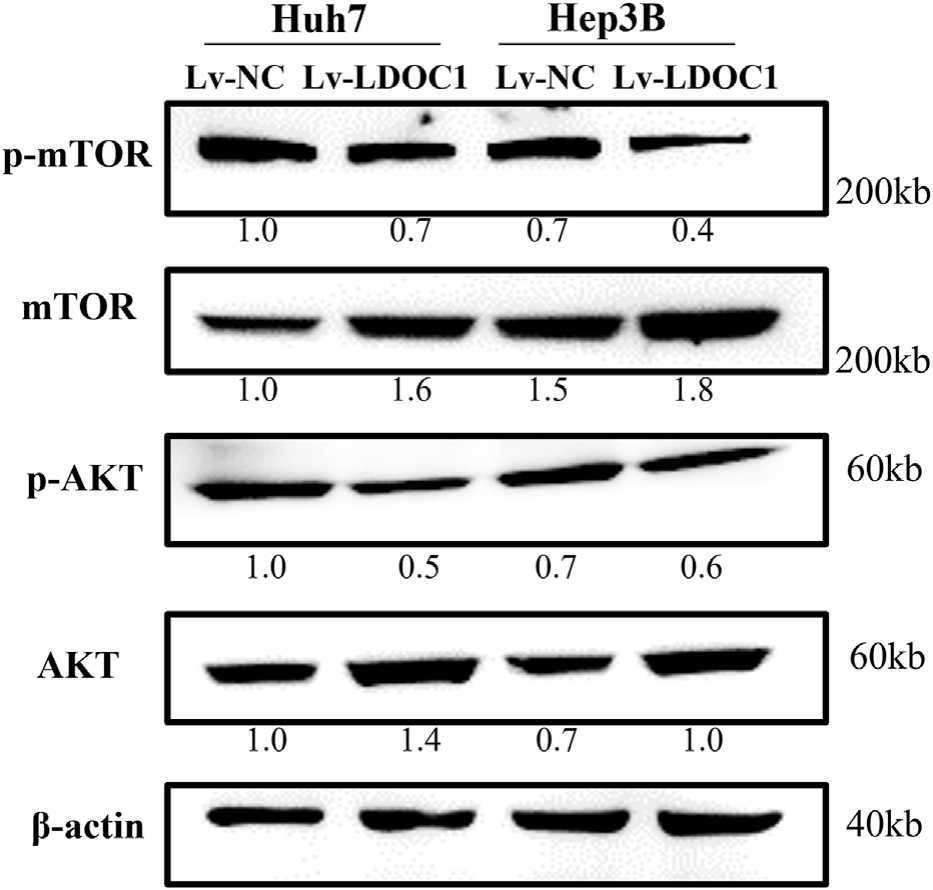
Western blot analysis found that Lv-LDOC1 increased the expression of total mTOR and total AKT and decreased the p-AKT/AKT and p-mTOR/mTOR.

To further explore the molecular mechanisms of LDOC1, we examined the expression of essential proteins in the AKT/mTOR signaling pathway implicated in cancer cell viability and proliferation. As shown in Figure 9, although the expression of total mTOR and total AKT were increased in the Lv-LDOC1 groups, the p-AKT/AKT and p-mTOR/mTOR were decreased, suggesting the activity of the AKT/mTOR pathway was downregulated in the Lv-LDOC1 groups compared to the Lv-NC groups.

## 4 Discussion

The interaction of biological and environmental factors contributes to the occurrence and progress of malignant tumors. However, from the point of view of molecular mechanism, the development of normal cells into cancer cells results from gene mutation and abnormal expression, including the activation of tumor-promoting genes and the inactivation of tumor-suppressor genes. Thus, identifying new HCC markers and their biological functions and molecular mechanisms will help in the diagnosis, treatment, and prognosis of HCC.

LDOC1 showed tumor-suppressed effects in several tumor cells (Nagasaki et al., 1999). Previous studies have found that the LDOC1 expression is reduced in certain malignancies, including colorectal cancer (Jiang et al., 2019), cervical cancer (Chen and Wang, 2019), pancreatic cancer (Nagasaki et al., 2003), prostate cancer (Salemi et al., 2016), and papillary thyroid carcinoma (Zhao et al., 2015). However, no reports have found an association between LDOC1 and HCC. Our study determined and validated the potential diagnostic and prognostic abilities of LDOC1. We identified that LDOC1 expression may be a valuable biomarker for HCC diagnosis. Moreover, the diagnostic performance was validated with qRT-PCR, IHC, and Western blot. In addition, the prognostic value of LDOC1 was evaluated with Kaplan-Meier Plotter, an online website. The results suggested that a higher level of LDOC1 predicted a favorable prognosis in the analyses of OS, PFS, RFS, and DSS.

Several studies have also reported the molecular mechanism by which LDOC1 regulates cancer cells. Jiang et al. demonstrated that LDOC1 overexpression inhibited cancer growth by downregulating the Wnt/β-catenin signaling in colorectal cancer (Jiang et al., 2019). Zhao et al. found that LDOC1 played a tumor suppressor role in papillary thyroid carcinoma by reducing the NF-κΒ signaling pathway (Zhao et al., 2015). In ovarian cancer, LDOC1 is downregulated through promoter methylation and may act as an early marker. All of those studies revealed that LDOC1 participated in the antitumor processes. Thus, we further performed functional experiments *via* over-expression of LDOC1 expression and determination of cell proliferation, colony formation, and migration ability. Our results demonstrated decreased cell proliferation, colony formation, and migration in the Huh7 and Hep3B cell lines after over-expressing LDOC1. Cell cycle assays also suggested that cell division was disordered after upregulating LDOC1, although the arrest stages vary in different cell lines. Previous studies have demonstrated that over-expressing LDOC1 contributed to G1 or G2 stage arrest (Jiang et al., 2019). In this study, we found LDOC1 up-regulation led to increased G1 and G2 stages in Huh7 while increased G2 stage in Hep3B. The apoptosis assays indicated that the LDOC1 overexpression led to an elevated apoptosis rate in both Huh7 and Hep3B cell lines. These results are consistent with the diagnostics and survival analyses. Therefore, we concluded that LDOC1 might act as a tumor suppressor gene in HCC.

The AKT/mTOR is an intracellular pathway that plays an important role in regulating cell proliferation and migration (Markowitsch et al., 2022; Shao et al., 2022; Zhang et al., 2022). Thus, the Western blot was used to explore the potential molecular effects of LDOC1 on liver cancer in this study. The results demonstrated that the p-AKT/AKT and p-mTOR/mTOR were down-regulated in both Huh7 and Hep3B cell lines, indicating decreased AKT/mTOR signaling activity.

However, this study has several limitations. For instance, the prognostic performance of LDOC1 for HCC requires cohort validation in the future. Moreover, further proof *in vivo* needs to be conducted to confirm the antitumor effects of LDOC1, although we have fully verified the LDOC1 impacts on the proliferation, colony formation, cell cycle, apoptosis, and migration of HCC cells *in vitro*.

## 5 Conclusion

In our study, LDOCl expression varied in tumor and nontumor tissue samples of HCC patients and was significantly low-expressing in tumor tissues. We further observed that the transcriptional levels for LDOC1 were associated with OS, PFS, RFS, and DSS. The elevated expression of LDOC1 led to a favorable prognosis, suggesting an antitumor effect of LDOC1 in HCC. In addition, the functional experiments by upregulating LDOC1 expression showed reduced cell proliferation, colony formation, and migration ability and increased apoptosis in Huh7 and Hep3B cell lines. Furthermore, we found that the biological function of LDOC1 was mediated *via* the downregulation of the AKT/mTOR pathway. In summary, our findings suggest that LDOC1 may have tumor-suppressive effects by inhibiting AKT/mTOR activation in HCC.

## Data Availability

The original contributions presented in the study are included in the article/supplementary material, further inquiries can be directed to the corresponding author.
